# Hsp70 facilitates trans-membrane transport of bacterial ADP-ribosylating toxins into the cytosol of mammalian cells

**DOI:** 10.1038/s41598-017-02882-y

**Published:** 2017-06-02

**Authors:** Katharina Ernst, Johannes Schmid, Matthias Beck, Marlen Hägele, Meike Hohwieler, Patricia Hauff, Anna Katharina Ückert, Anna Anastasia, Michael Fauler, Thomas Jank, Klaus Aktories, Michel R. Popoff, Cordelia Schiene-Fischer, Alexander Kleger, Martin Müller, Manfred Frick, Holger Barth

**Affiliations:** 1 Institute of Pharmacology and Toxicology, University of Ulm Medical Center, 89081 Ulm, Germany; 2 Department of Internal Medicine I, University of Ulm, 89081 Ulm, Germany; 3 Institute of General Physiology, University of Ulm, 89081 Ulm, Germany; 4 Institute of Experimental and Clinical Pharmacology and Toxicology, University of Freiburg, 79104 Freiburg, Germany; 5 Department of Anaerobic Bacteria, Pasteur Institute, 75015 Paris, France; 6 Institute for Biochemistry and Biotechnology, Martin Luther University Halle-Wittenberg, 06120 Halle (Saale), Germany

## Abstract

Binary enterotoxins *Clostridium* (*C*.) *botulinum* C2 toxin, *C. perfringens* iota toxin and *C. difficile* toxin CDT are composed of a transport (B) and a separate non-linked enzyme (A) component. Their B-components mediate endocytic uptake into mammalian cells and subsequently transport of the A-components from acidic endosomes into the cytosol, where the latter ADP-ribosylate G-actin resulting in cell rounding and cell death causing clinical symptoms. Protein folding enzymes, including Hsp90 and peptidyl-prolyl *cis/trans* isomerases facilitate transport of the A-components across endosomal membranes. Here, we identified Hsp70 as a novel host cell factor specifically interacting with A-components of C2, iota and CDT toxins to facilitate their transport into the cell cytosol. Pharmacological Hsp70-inhibition specifically prevented pH-dependent trans-membrane transport of A-components into the cytosol thereby protecting living cells and stem cell-derived human miniguts from intoxication. Thus, Hsp70-inhibition might lead to development of novel therapeutic strategies to treat diseases associated with bacterial ADP-ribosylating toxins.

## Introduction

*Clostridium* (*C*.) *perfringens* iota toxin, *C*. *botulinum* C2 toxin and *C*. *difficile* toxin CDT belong to the group of binary actin ADP-ribosylating toxins causing severe enterotoxicity in humans and animals^1–4^. They are secreted by bacteria as two non-linked proteins, which interact on the surface of target cells. The binding/translocation (B) component facilitates uptake of the enzymatic active (A) component into the host cell cytosol, where the A-components mono-ADP-ribosylate G-actin^5–7^. This results in depolymerization of actin filaments and cell-rounding^8–11^, which is responsible for destruction of the gut barrier and causing of clinical symptoms, i.e. enterotoxicity.

Cellular uptake of C2 toxin, the prototype of this toxin family, has been studied in detail. Proteolytic activation of the B-component C2II (~80/100 kDa, dependent on the strain^12^) results in biologically active C2IIa (~ 60/80 kDa)^12, 13^. C2IIa forms heptameric complexes that bind to an asparagine-linked carbohydrate structure, which is present on the surface of all cell types^13–16^. The A-component C2I (~49 kDa) binds to the C2IIa-heptamer and the C2IIa/C2I complexes are internalized by receptor-mediated endocytosis. Acidification of endosomes by a vesicular ATPase (v-ATPase) leads to conformational changes of C2IIa, which then inserts into endosomal membranes and forms trans-membrane pores for the transport of C2I into the cytosol^13^. C2I unfolds to translocate through C2IIa pores^17, 18^.

The uptake of iota toxin is widely comparable (for review see ref. 19). The heptameric binding/translocation component Ib facilitates the uptake and translocation of the enzymatic active Ia into the cytosol^20^. CDT is closely related to iota toxin (82% homology between activated Ib and CDTb) and its uptake mechanism is comparable to the iota toxin^21, 22^. Moreover, iota and CDT share the same cell surface receptor, the lipolysis-stimulated lipoprotein receptor (LSR)^23, 24^ and exploit in addition to LSR CD44 for uptake^25^.

Despite these differences between C2 toxin and iota-like toxins, a common membrane translocation mechanism involving requirement of the host cell chaperone Hsp90 and peptidyl-prolyl *cis/trans* isomerases (PPIases) of the cyclophilin (Cyp) and FK506-binding protein (FKBP) families is evident (refs 26–31 for review see ref. 32). Recently, we discovered that, in addition to Hsp90 and PPIases, the heat shock protein Hsp70 facilitates the trans-membrane transport of iota toxin into the host cell cytosol^33^. Hsp70 also facilitates the translocation of proteins across intracellular membranes for example in mitochondria or the ER^34, 35^. Moreover, Hsp70 is part of Hsp90-containing multi-chaperone complexes that facilitate the folding and activation of steroid hormone receptors^36–38^. This is particularly interesting given our previous results that Hsp90 and further members of the multi-chaperone complex, i.e. Cyp40 and FKBP51, are required for the membrane translocation of iota, C2 and CDT toxins. Therefore, we investigated whether Hsp70 also plays a role during the uptake of other clostridial binary toxins, i.e. C2 and CDT toxins. To this end, we used two specific pharmacological inhibitors of Hsp70 activity. VER-155008 (VER) binds to the N-terminal located ATP-binding pocket of Hsp70 and the constitutive form Hsc70, thereby inhibiting its folding activity^39^. The novel inhibitor HA9 is specific for only Hsp70 and targets its C-terminal substrate binding domain resulting in impaired binding of client proteins^33^. Our results demonstrate that VER and HA9 both inhibit the membrane translocation of iota, C2 and CDT toxins and, therefore, lead to an impaired intoxication of cells and stem-cell derived human intestinal organoids (miniguts). By performing fluorescence microscopy, we demonstrate for the first time that the enzyme components of these toxins interact with Hsp70 in the cytosol of living cells, indicating the importance of Hsp70 for efficient uptake of clostridial binary toxins into the host cell cytosol.

## Results

### Enzyme components of iota, C2 and CDT toxins directly and specifically bind to Hsp70 and Hsc70 *in vitro*

**Figure 1:**
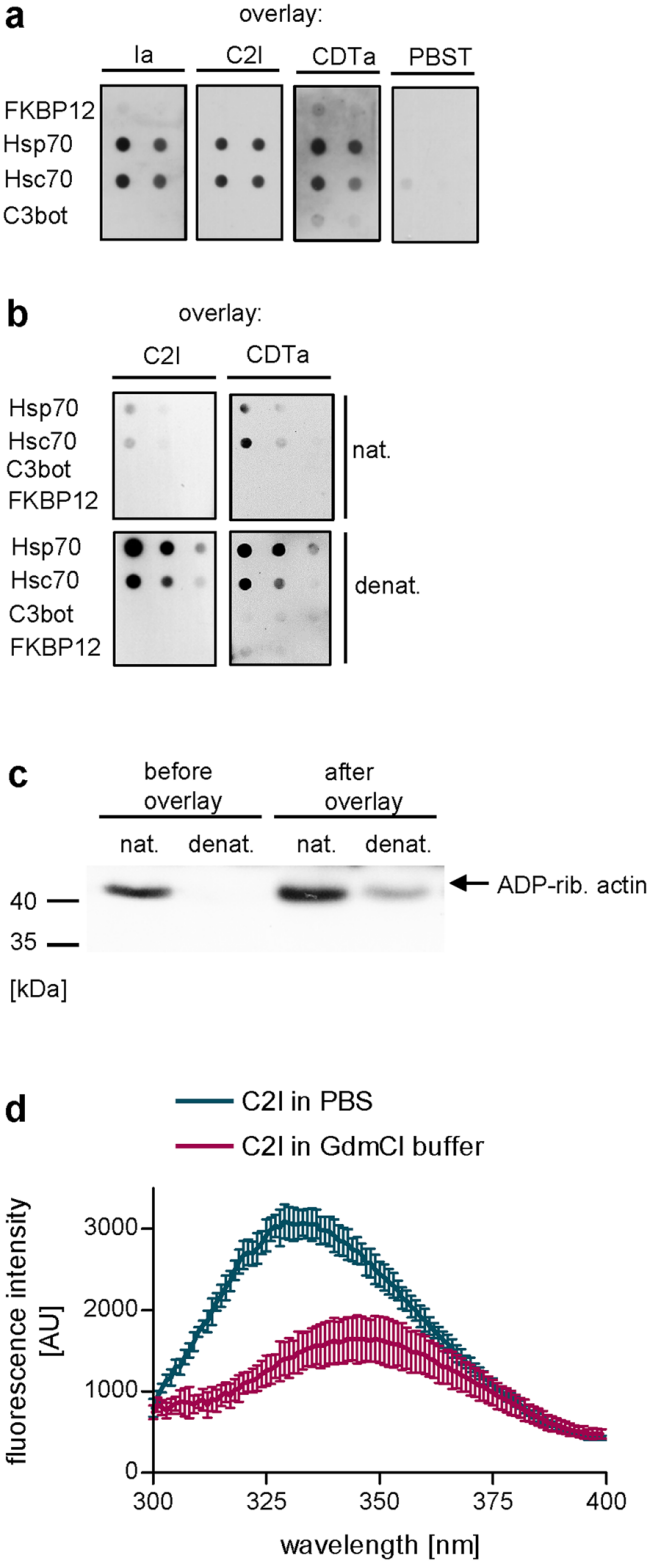
(**a**) Hsp70 and Hsc70 directly bind to C2I, Ia and CDTa *in vitro*. Decreasing concentrations of recombinant Hsp70 and Hsc70 were spotted onto a membrane using the dot blot system. For control, FKBP12 and *C. botulinum* C3 protein were spotted. Subsequently, the membrane was blocked, cut and probed with biotin-labeled proteins C2I, Ia, CDTa (200 ng/ml) or with PBST for control. Bound C2I, Ia and CDTa were detected with Strep-POD using the ECL system. Equal amounts of spotted protein was confirmed by Ponceau S staining (not shown). The dot blot panel was cropped for presentation purposes only. (**b**) Denaturation of enzyme components enhances binding to Hsc/Hsp70. The experiment was conducted as described in (**a**) except that enzyme components were denatured or left untreated for native conformation prior to overlay. The dot blot panel was cropped for presentation purposes only. (**c**) Denaturation of C2I is confirmed by loss of enzyme activity. Samples from overlay solutions prepared in (**b**) were taken prior and after 1 h overlay incubation and tested for *in vitro* enzyme activity. Therefore, samples were incubated with cell lysate in the presence of biotin-NAD^+^. Biotinylated i.e. ADP-ribosylated actin was detected by Western blotting. Ponceau S staining demonstrates equal amounts of protein (not shown). The Western blot panel was cropped for presentation purposes only. (**d**) Conformational changes of C2I induced by GdmCl. Tryptophan fluorescence scan was performed to measure conformational changes of C2I by GdmCl compared to PBS.

Prompted by our previous results that Ia and C2I bind to Hsp70 and Hsc70 *in vitro* ^33^, we performed dot blot analysis to confirm that the enzyme component CDTa of CDT also binds to recombinant Hsp/c70 (Fig. 1a). In line with previous results, the enzyme components do not bind to the C3bot protein of *C. botulinum* (used as control) or FKBP12, a small FKBP isoform of the PPIase family, demonstrating the specificity of this binding. Interestingly, the denatured, i.e. partially unfolded, enzyme components displayed enhanced binding to Hsp/c70 compared to their native conformations as demonstrated for C2I and CDTa in Fig. 1b and for Ia recently^33^. The unfolding/denaturation of the enzyme component was demonstrated for the prototypic C2I by monitoring of enzyme activity *in vitro* (Fig. 1c) as performed before^31^. At the beginning of the overlay incubation loss of enzyme activity was observed. Although after 1 h of incubation, enzyme activity of the denatured C2I was detected, it was still significantly reduced compared to the native C2I suggesting that the majority of enzyme molecules were in the denatured/unfolded conformation. Moreover, changes in tryptophan fluorescence spectra were observed when C2I was incubated in denaturing GdmCl buffer compared to C2I in PBS, indicating conformational changes, most likely unfolding of C2I due to the known denaturing effect of GdmCl (Fig. 1d).

### Pharmacological inhibition of Hsp70 activity inhibits intoxication of cells with iota, C2 and CDT toxins

**Figure 2:**
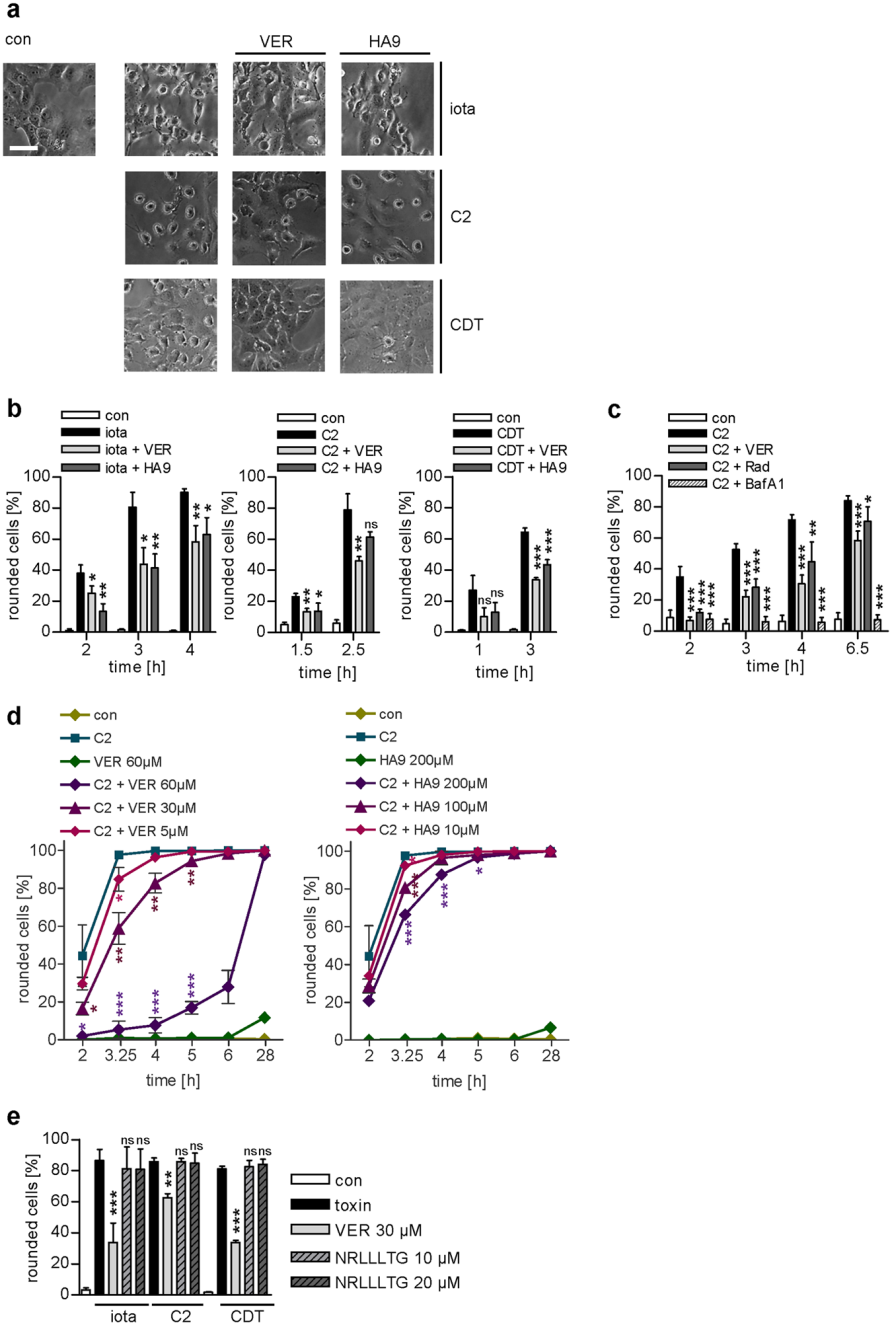
The Hsc/Hsp70 inhibitor VER and the specific Hsp70 inhibitor HA9 inhibit intoxication of Vero cells with iota, C2 and CDT toxin. Vero cells were pre-incubated for 30 min at 37 °C with VER (30 µM) or HA9 (100 µM) or left untreated for control. Subsequently, iota toxin (15 ng/ml Ia plus 30 ng/ml Ib), C2 toxin (50 ng/ml C2I plus 100 ng/ml C2IIa) and CDT (15 ng/ml CDTa plus 30 ng/ml CDTb) were added. (**a**) Pictures show the toxin-induced morphological changes after 3 h for iota toxin, after 2.5 h for C2 toxin and after 1 h for CDT intoxication. Bar = 50 µm. (**b**) The percentage of rounded cells was determined from images at the indicated time points. Values are given as mean ± SD (n = 3). Significance was tested by using the Student’s t test and values refer to samples treated with toxin only (ns = not significant, *p < 0.05, **p < 0.01, ***p < 0.001). (**c**) Inhibition of proteasomal degradation does not affect inhibitory effect of VER on C2 intoxication. Vero cells were pre-incubated for 1 h with the proteasome inhibitor MG132 (30 µM). VER (30 µM), Rad (20 µM) and BafA1 (100 nM) were added for additional 30 min. Subsequently, cells were challenged with C2 toxin (50 ng/ml C2I plus 100 ng/ml C2IIa). Pictures were taken and analyzed as described in (**b**). (**d**) Concentration-dependent inhibition of C2 toxin intoxication by VER and HA9. Cells were treated as described in (**b**) 250 ng/ml C2I plus 500 ng/ml C2IIa were used. (**e**) Non-cell permeable Hsp70-inhibitory peptide NRLLLTG does not inhibit intoxication with iota, C2 or CDT toxins. Vero cells were pre-incubated with NRLLLTG (10 or 20 µM) for 30 min and then challenged with the toxins for 2 h. Rounded cells were counted as described above. Values are given as mean ± SD (n = 3). Significance was tested by using the Student’s t test and values refer to samples treated with toxin only (ns = not significant, *p < 0.05, **p < 0.01, ***p < 0.001).

Hsp70 inhibitors VER and HA9 both markedly delayed the intoxication of Vero cells with iota, C2 and CDT in a time- and concentration-dependent manner (Fig. 2a–d). Rounding of adherent cells is the consequence of G-actin modification and indicates a highly specific endpoint of intoxication and of cytosolic uptake of the A-components. Quantification of rounded cells clearly indicated the inhibitory effect of VER and HA9 on intoxication with iota, C2 and CDT toxins (Fig. 2b). In contrast to the intracellularly acting VER and HA9, the cell-impermeable peptide NRLLLTG, which is known to compete with Hsp70 client proteins for binding to the Hsp70 substrate binding domain, had no protective effect on intoxication of Vero cells with iota, C2 and CDT toxins (Fig. 2e), suggesting that cytosolic and not extracellular Hsp70 plays a role for the mode of action of these toxins. Moreover, if the proteasomal degradation was inhibited in cells by MG132 and then VER was added a comparable inhibitory effect of VER on C2-intoxication was observed (Fig. 2c). This suggests that inhibition of intoxication by VER is probably not due to enhanced proteasomal degradation of cytosolic enzyme component upon Hsp70-inhibition. The inhibitory effect of the Hsp90-inhibitor radicicol (Rad) was also not impaired if the proteasomal degradation was inhibited by MG132 (Fig. 2c), which we also reported earlier^27^.

### VER and HA9 affect the ADP-ribosylation status of actin in cells but do not inhibit the enzyme activity of the toxins

**Figure 3:**
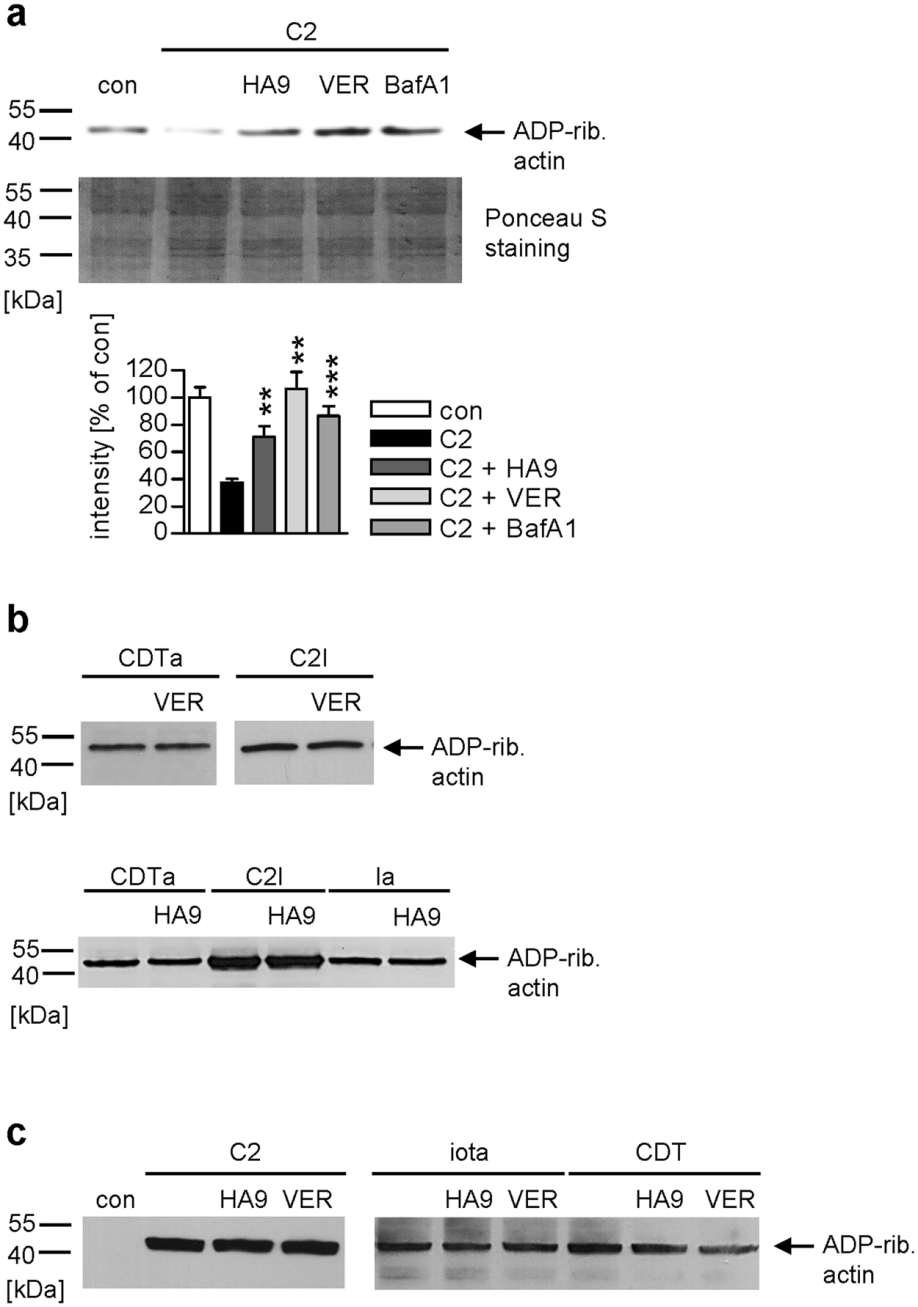
(**a**) Effect of VER and HA9 on the ADP-ribosylation of actin in C2 toxin-treated cells. Vero cells were pre-incubated with VER (30 µM) or HA9 (100 µM) for 30 min at 37 °C. For control, cells were pre-incubated with BafA1 (30 nM) or left untreated. Subsequently, C2 toxin (75 ng/ml C2I plus 150 ng/ml C2IIa) was added for 4 h at 37 °C. Then, cells were lysed and all samples incubated with fresh C2I (300 ng) and biotin-NAD^+^ for 30 min at 37 °C. Biotinylated, i.e. ADP-ribosylated (ADP-rib.) actin was detected by Western blotting and intensity of signals quantified by densitometry. Values show the percentage of signal referring to untreated cells (con) and are normalized on the amount of whole lysate protein and are given as mean ± SEM (n = 4). Significance was tested by using the Student’s t test and values refer to samples treated with toxin only (**p < 0.01, ***p < 0.001). The Western blot panel was cropped for presentation purposes only. (**b**) VER and HA9 do not inhibit enzyme activity of iota, C2 or CDT toxin. Vero cell lysate was pre-incubated with 30 µM VER or 100 µM HA9 for 30 min at 37 °C. For control, cell lysates were left untreated. Subsequently, C2I, Ia or CDTa (each 200 ng) and biotin-NAD^+^ were added for 20 min at 37 °C. Biotin-labeled i.e. ADP-ribosylated actin was detected as described before. The Western blot panel was cropped for presentation purposes only. (**c**) VER and HA9 do not inhibit the receptor binding of iota, C2 or CDT toxin. After pre-incubation with HA9 (100 µM) or VER (30 µM), Vero cells were cooled to 4 °C and C2 toxin (100 ng/ml C2I plus 200 ng/ml C2IIa), iota toxin (100 ng/ml Ia plus 200 ng/ml Ib) and CDT (100 ng/ml CDTa plus 200 ng/ml CDTb) were added for 30 min. For control, cells were left untreated. After washing, the cell-bound C2, iota and CDT toxin were detected by analyzing the ADP-RT activity of the cell-associated C2I, Ia and CDTa *in vitro* via biotin-NAD^+^ and Western blotting. The Western blot panel was cropped for presentation purposes only.

After intoxication of Vero cells with the prototypic C2 toxin in the presence or absence of VER/HA9, lysates of these cells were incubated with fresh C2I in the presence of biotin-labeled co-substrate NAD^+^. During this incubation, G-actin, which was not modified during incubation of living cells with C2 toxin, became ADP-ribosylated *in vitro* resulting in biotin-labeled G-actin that was detected by Western blotting (Fig. 3). The results clearly demonstrate that inhibition of Hsp70 with VER or HA9 strongly reduced C2-mediated ADP-ribosylation in living cells. Actin from untreated cells gives a strong signal after *in vitro* ADP-ribosylation while actin from C2 toxin-treated cells gives a weak signal because most of this actin was already ADP-ribosylated by C2 toxin in living cells (Fig. 3a). In the presence of VER or HA9 during C2-intoxication less actin was ADP-ribosylated in living cells compared to cells treated with C2 toxin in the absence of an inhibitor (Fig. 3a). As control, bafilomycinA1 (BafA1) was included. BafA1 prevents endosome acidification and transport of C2I from endosomes into the cytosol. Densitometric quantification also displayed this protective effect of VER, HA9 or BafA1 (Fig. 3a). However, from these results it could not be concluded whether less C2I protein or less active C2I reached the cytosol in the presence of the Hsp70 inhibitors, or whether enzymatically C2I was probably there but VER and HA9 inhibited the ADP-ribosylation of the actin per se. As shown in Fig. 3b, VER or HA9 did not decrease the ADP-ribosylation of actin by C2I, Ia or CDTa *in vitro*. Thus, VER and HA9 likely interfere with the toxin transport into the cytosol.

### VER and HA9 inhibit pH-dependent transport of the A-components of clostridial binary toxins across membranes

**Figure 4:**
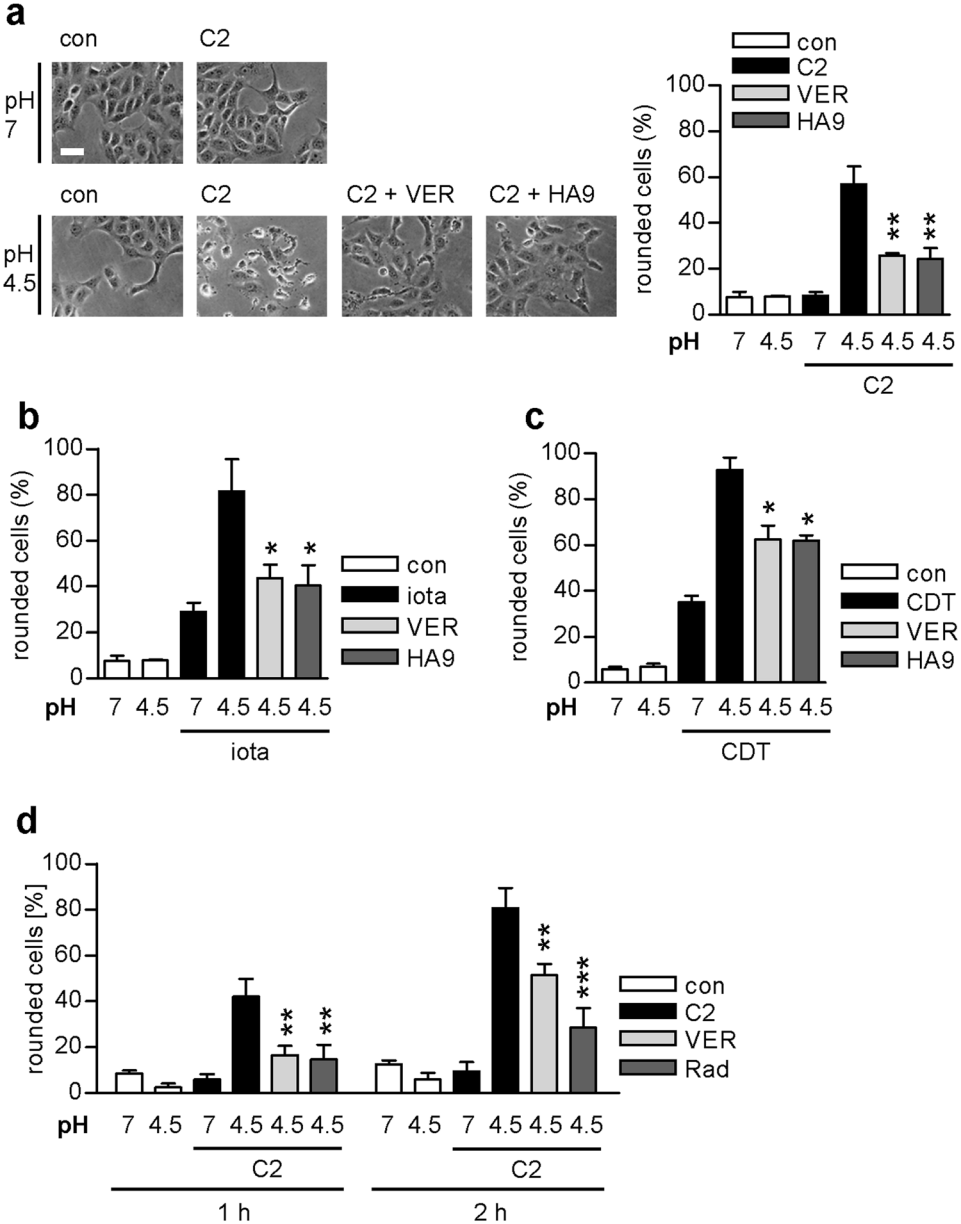
Pharmacological inhibition of Hsp/Hsc70 activity inhibits the pH-dependent membrane translocation of the enzyme components of iota, C2 and CDT toxin into the cytosol. For the toxin translocation assay, Vero cells were pre-incubated with HA9 (100 µM) or VER (30 µM) in combination with BafA1 (100 nM) and for control with BafA1 (100 nM) alone for 30 min at 37 °C. After binding of C2 toxin (100 ng/ml C2I plus 200 ng/ml C2IIa) (**a**), iota toxin (200 ng/ml Ia plus 200 ng/ml Ib) (**b**), or CDT toxin (150 ng/ml CDTa plus 150 ng/ml CDTb) (**c**) at 4 °C, cells were challenged with warm acidic medium to allow the direct translocation of the enzyme components across the cytoplasmic membrane into the host cell cytosol. For control, cells were incubated with neutral medium. Subsequently, cells were further incubated at 37 °C and cell morphology was monitored. The percentage of rounded cells was determined from images taken after 2 h for C2 and iota toxin and after 1.5 h for CDT. (**d**) Inhibition of pH-dependent membrane translocation of C2I by VER is not impaired by inhibition of proteasomal degradation. Vero cells were pre-incubated with MG132 (30 µM) for 1 h. Subsequently, the experiment was conducted as described in (**a**). 100 ng/ml C2I and 200 ng/ml C2IIa were used. Values are given as mean ± SD (n = 3). Bar = 50 µm. Significance was tested by using the Student’s t test and values refer to samples treated with toxin only under acidic conditions (ns = not significant, *p < 0.05, **p < 0.01, ***p < 0.001).

VER and HA9 did not inhibit binding of C2, iota or CDT toxins to cells, as detected by measuring the enzyme activity of the respective cell-bound toxin (Fig. 3c). Next, the effect of VER and HA9 on the pH-dependent membrane translocation was tested. To this end, we investigated the translocation of cell-bound toxin across the cytoplasmic membrane of living cells after pH-shift. Therefore, Vero cells were pre-incubated with BafA1 to block normal toxin uptake^13^ and some cells were in addition incubated with VER or HA9. All cells were then cooled to 4 °C and the respective toxin was added allowing receptor binding but not internalization. Warm and acidic medium was added to induce pore formation by their B-components into the cytoplasmic membrane and translocation of their enzyme components through the pores into the host cell cytosol. Toxin-induced cell rounding only occurred when toxin-treated cells were exposed to acidic medium, i.e. when C2I translocated across the cytoplasmic membrane due to the acidic pulse (Fig. 4a). Fewer cells rounded up after pre-treatment with VER or HA9 compared to cells treated with toxin only (Fig. 4a) suggesting that Hsp70 facilitates the pH-dependent trans-membrane transport of the A-components into the cytosol of mammalian cells. Comparable results were obtained for iota toxin and CDT (Fig. 4b,c). The inhibition of proteasomal degradation by MG132 did not result in the loss or impairment of the inhibitory effect due to Hsp70-inhibition during the pH-triggered trans-membrane transport of the enzyme component (Fig. 4d). This result also suggests that the reason underlying the inhibited intoxication is not an enhanced degradation of the enzyme component in the cytosol if Hsp70 activity is inhibited in the cells.

**Figure 5:**
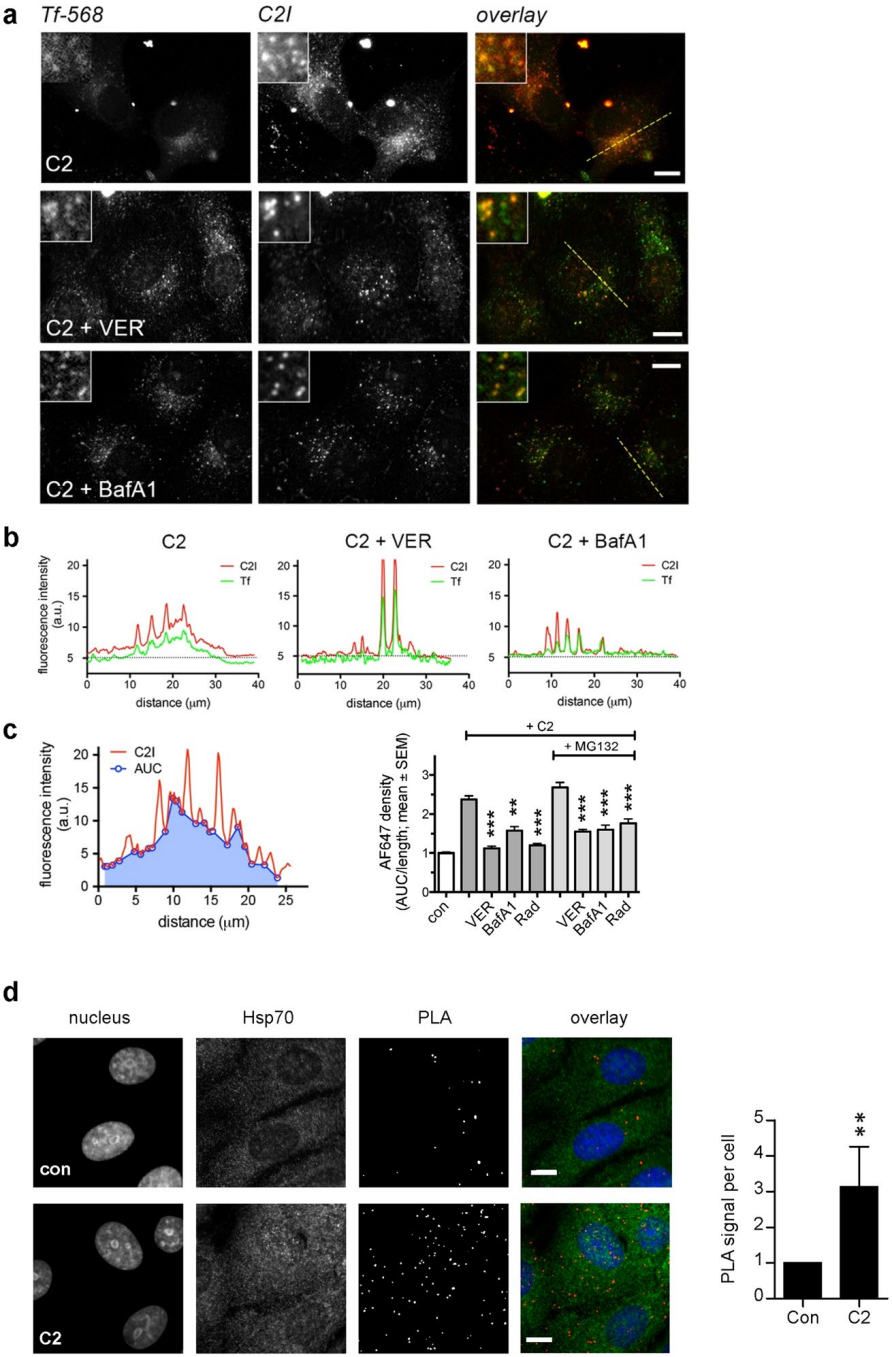
Hsp70 inhibition by VER leads to enhanced co-localization of C2I with early endosomes and a reduced amount of C2I in the cytosol of target cells. (**a**) Vero cells were pre-incubated with 30 µM VER, 100 nM BafA1 or left untreated. Subsequently, cells were challenged with an enzymatic inactive C2 toxin mutant (1 µg/ml C2IE387/389Q plus 1 µg/ml C2IIa) in the presence of 10 µg/ml transferrin-Alexa Fluor 568 for 1 h at 37 °C. C2I and transferrin i.e. early endosomes were visualized by fluorescence microscopy. Bars = 10 μm. (**b**) Plot profiles along the dotted lines in (**a**) illustrate increased C2I intensity in Tf-568 positive structures and that the diffuse cytosolic labeling is reduced in cells treated with either VER or BafA1. (**c**) Quantitative analysis of the average cytosolic C2I signal, excluding endosomal peaks. Areas under the curve (AUCs) were calculated and corrected for length of the line (AUC/length) resulting in the cytosolic C2I density. The experiment was also performed with an additional pre-incubation of 1 h with 30 µM MG132, an inhibitor of proteasomal degradation. Data represent means from 25–130 cells per condition form 2–5 independent replicates. (**d**) C2I interacts with Hsp70 in cultured cells. Vero cells were incubated with C2 toxin (1 µg C2I plus 1 µg C2IIa) or left untreated for control for 1 h at 37 °C. Subsequently, the fluorescence-based PLA assay was performed according to the manufacturer’s manual. The nucleus was stained with Hoechst. PLA signal represents one protein interaction event of C2I and Hsp70. Bars = 10 µm. The amount of PLA signals per nucleus was quantified by a software using a computer-vision algorithm based on OpenCV. Values are normalized to control samples and are given as mean ± SEM (n = 5 independent experiments, at least 100 cells per sample per experiment were analyzed). Significance was tested by using the Mann-Whitney test (**p < 0.05).

The finding that less C2I reaches the host cell cytosol when Hsp70 is inhibited was confirmed and extended by an alternative approach using fluorescence microscopy (Fig. 5a–c). After incubation of cells with C2 and fluorescent transferrin (Tf-568), C2I co-localized with Tf-568 in bright fluorescent punctae indicating endosomal localization of C2I. In addition, some C2I was visible as diffuse, cytosolic staining. VER treatment, however, resulted in a markedly reduced cytosolic signal, comparable to results obtained with BafA1 as a positive control (Fig. 5a,b). Quantification of the C2I-derived cytosolic fluorescence signal confirmed that inhibition of Hsp70 results in a reduced amount of C2I protein in the cytosol (Fig. 5c). A comparable effect was observed for the Hsp90 inhibitor Rad (Fig. 5c). The role of Hsp90 for toxin translocation and the inhibitory effect of Rad on intoxication with ADP-ribosylating toxin has been demonstrated for C2, iota and CDT toxins in detail earlier^27, 28, 30^. The effect of inhibition of the proteasomal degradation was also investigated in this assay (Fig. 5c). The results clearly indicate that less enzyme component can be detected in the cytosol of VER-treated cells even if the proteasomal degradation is inhibited by MG132. This demonstrates that the translocation from endosomes into the cytosol is impaired upon Hsp70-inhibiton and excludes that less C2I molecules are detected in the cytosol due to misfolding and subsequent rapid degradation of C2I. Comparable results were obtained for BafA1 employed as a positive control and the Hsp90-inhibitor Rad. Taken together, the results obtained by fluorescence microscopy clearly indicate that more C2I remained in early endosomes and did not translocate into the cytosol when Hsp70 was inhibited supporting our findings from the pH-shift translocation assay.

### C2I interacts with Hsp70 in cultured cells

The requirement of host cell chaperones/PPIases for cellular uptake of enzyme subunits of various bacterial ADP-ribosylating toxins has been well established and the binding of these host cell factors to the ADP-ribosyltransferase (ADP-RT) subunits was clearly demonstrated *in vitro* (refs 26, 30, 31, 33 for review see ref. 40). However, an interaction for any of these host cell factors with an enzyme component of ADP-ribosylating toxins has not yet been observed in cells directly. Here, we demonstrate the interaction between C2I and Hsp70 in cells, using the fluorescence-based proximity ligation assay (PLA) (Fig. 5d). Therefore, cells were incubated with or without C2 toxin and then probed with antibodies against C2I and Hsp70. Secondary antibodies containing oligonucleotide sequences were applied and if they got in close proximity, ligation and rolling circle amplification resulted in fluorescence labeling (PLA signal), representing the interaction of C2I and Hsp70. Figure 5d shows that the PLA signal (red) was significantly enhanced in cells treated with C2 toxin compared to control cells without toxin, clearly indicating the interaction of Hsp70 with C2I in these cells. Proper binding and specificity of the primary antibodies was confirmed by additional incubation of samples with fluorescence labeled secondary antibodies directly visualizing C2I (not shown) and Hsp70. Hsp70 staining indicates that PLA signal is associated with cells. Moreover, if one of the required antibodies (anti-C2I, anti-Hsp70 and two PLA antibodies) was left out, the PLA signal was on background level (data not shown), comparable to the signal in control samples. Quantification of PLA signals per nucleus also clearly displayed an interaction signal for C2I and Hsp70 interaction (Fig. 5d).

### Hsp70 activity is required for uptake of an isolated ADP-RT domain

**Figure 6:**
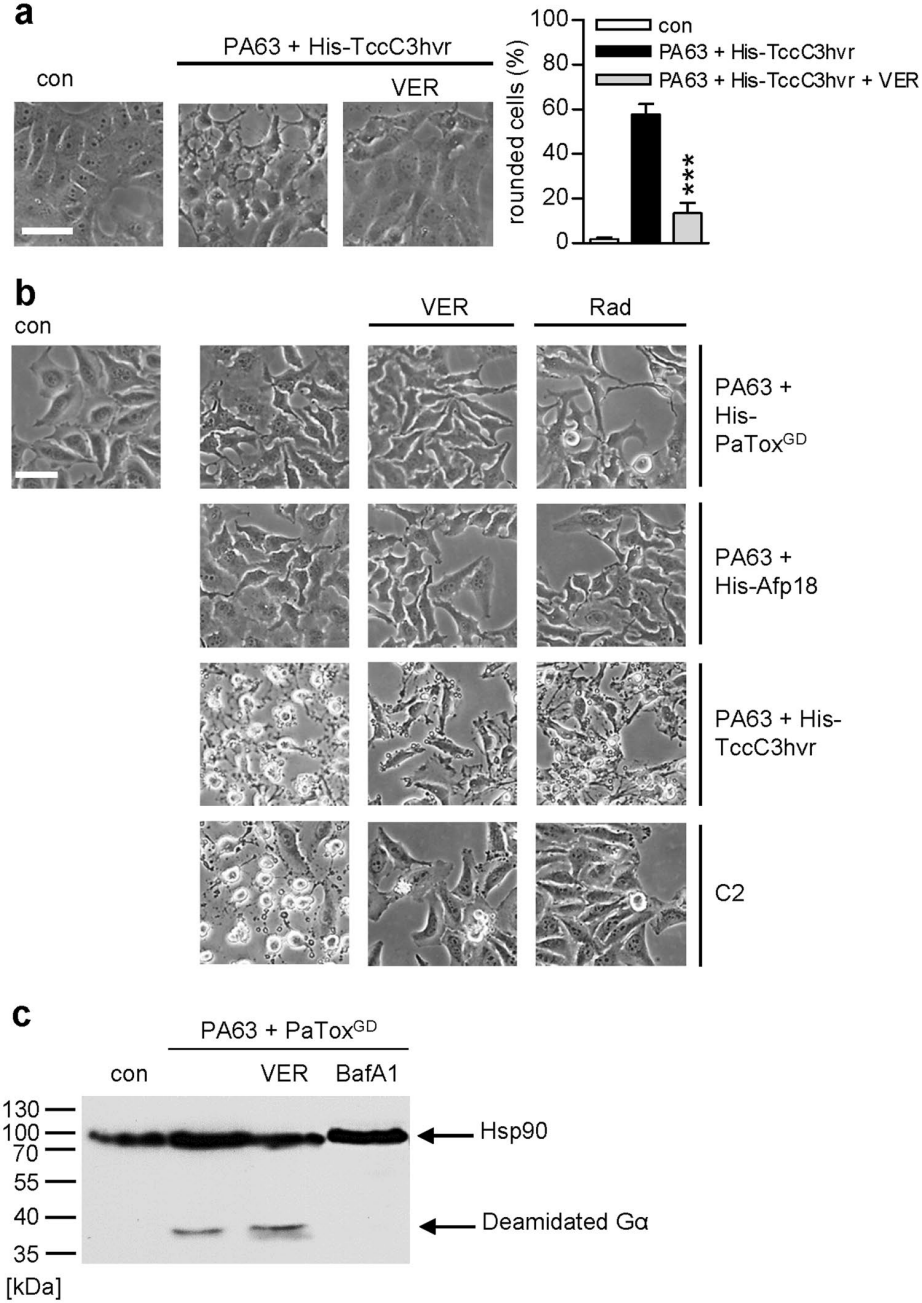
VER interferes with the uptake of an isolated ADP-RT domain (**a**) but has no inhibitory effect on uptake of isolated glycosyltransferases or deamidases (**b**). (**a**) Vero cells were pre-incubated with VER (30 µM) for 30 min and then challenged with PA63 (100 ng/ml) plus His-TccC3hvr (100 ng/ml) for 3 h at 37 °C. The percentage of rounded cells was determined. Values are given as mean ± SD (n = 3). Bar = 50 µm. Significance was tested by using the Student’s t test and values refer to samples treated with toxin only (***p < 0.001). (**b**) HeLa cells were pre-incubated with 30 µM VER (second lane), 20 µM Rad (third lane) or left untreated for negative control (=con) and positive control (first lane, cells treated with toxin only) for 30 min. Subsequently, cells were challenged with 500 ng/ml PA63 plus 370 ng/ml His-PaToxGD (first row) or 500 ng/ml PA63 plus 370 ng/ml His-Afp18 (second row) for 1 h or with 200 ng/ml PA63 plus 100 ng/ml His-TccC3hvr (third row) or 50 ng/ml C2I plus 100 ng/ml C2IIa (fourth row) for 3 h at 37 °C. Bar = 50 µm . **(c)** VER has no inhibitory effect on the deamidation status of cells treated with PA63 plus His-PaTox^GD^. HeLa cells were pre-incubated with VER (30 µM) or BafA1 (30 nM) or left untreated for control. Subsequently, 125 ng/ml PA63 plus 93 ng/ml His-PaTox^GD^ were added. After 4 h, cells were washed, lysed and samples were subjected to SDS-PAGE. In Western Blot analysis, the modified, i.e. deamidated, Gα was detected with a deamidation-specific antibody (anti-GαQ205). Equal amounts of protein were confirmed by Hsp90 detection. The Western blot panel was cropped for presentation purposes only.

Previous work suggests that requirement of Hsp90/PPIases for membrane translocation might be specific and characteristic only for toxins with ADP-RT activity. Here, we demonstrate that inhibition of Hsp70 activity by VER protects Vero cells from intoxication with the isolated ADP-RT domain His-TccC3hvr of the *Photorhabdus* (*P*.) *luminescens* PTC3 toxin when His-TccC3hvr was transported into the cytosol via PA63, as determined by the analysis of rounded cells (Fig. 6). PA63, the binding/translocation component of the *Bacillus* (*B*.) *anthracis* toxin, is able to deliver His-tagged proteins via receptor-mediated endocytosis and translocation from acidic endosomes into the cytosol^41–45^. However, uptake of other His-tagged bacterial enzymes, which are not ADP-RTs, like the glycosyltransferase and deamidase domain of the *P. asymbiotica* protein toxin (His-PaTox^GD^) or the glycosyltransferase domain of Afp18 (His-Afp18) from *Yersinia ruckeri*, was not inhibited by VER or Rad (Fig. 6b). PaTox and Afp18 both modify G proteins resulting in actin cytoskeleton re-organization indicated by morphological changes (Fig. 6b). VER or Rad did not prevent toxin-induced changes in cell morphology. Moreover, VER had no inhibitory effect on deamidation of Gα protein in cells treated with PA63 plus PaTox^GD^ whereas BafA1 inhibits deamidation as described before (Fig. 6c)^43^. In the same experiment, VER and Rad inhibited intoxication of cells with C2 toxin (Fig. 6b). These results further support the hypothesis of a common chaperone/PPIase-dependent translocation mechanism specific for ADP-ribosylating toxins and implicate that this specificity includes Hsp70 requirement.

### VER has a protective effect on human intestinal organoids upon intoxication with CDT

**Figure 7:**
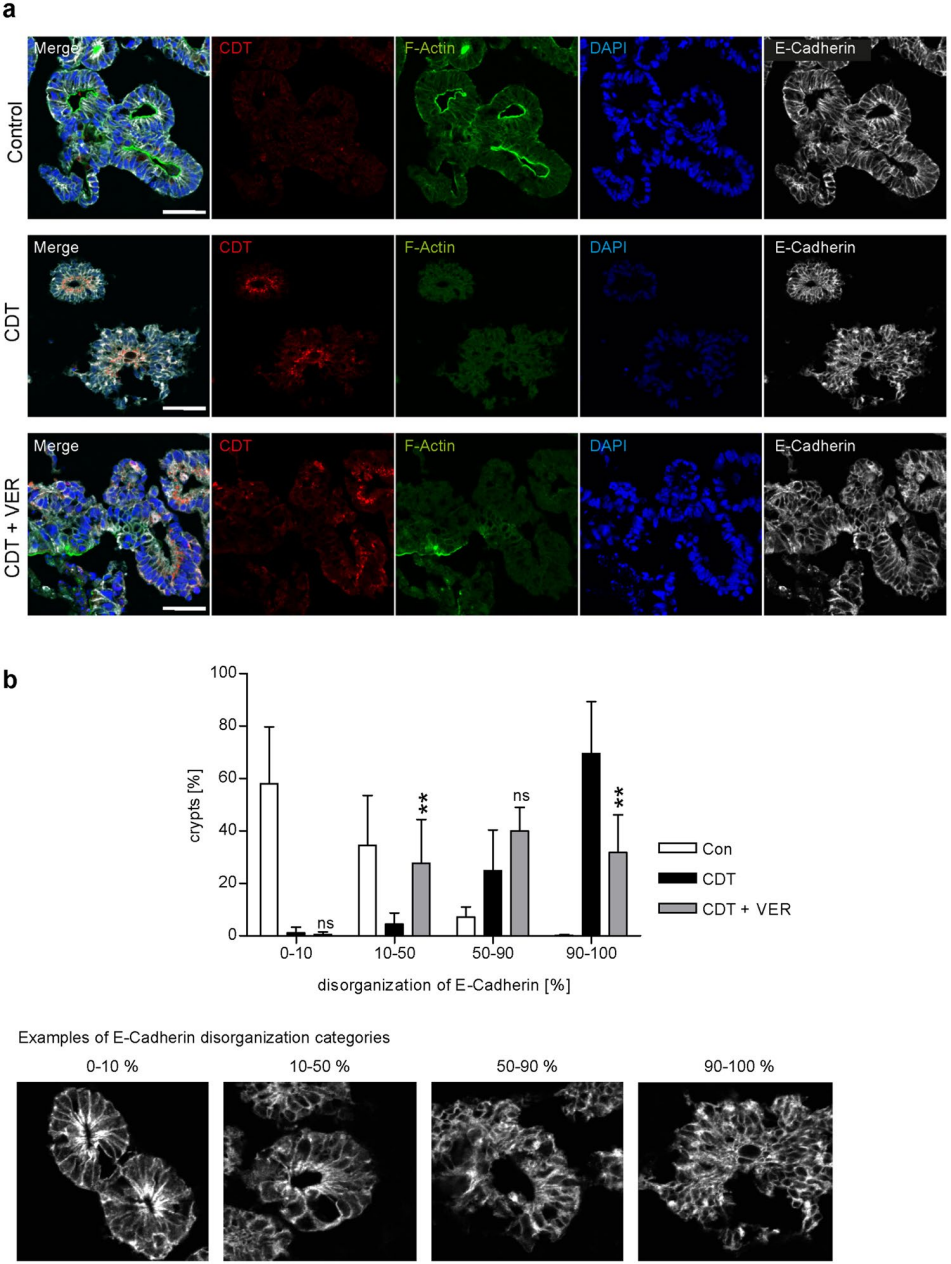
VER reduces the CDT-induced destruction of F-actin and disorganization of E-cadherin in human miniguts. (**a**) Miniguts were pre-incubated for 30 min at 37 °C with 60 µM VER or left untreated for controls. CDTa (500 ng/ml) plus CDTb (1000 ng/ml) were added for 3 h. After washing and extraction from matrigel, miniguts were fixed and frozen sections were prepared. CDTa, E-cadherin, nuclei and F-actin were stained and visualized by confocal microscopy. Bar = 50 µm. (**b**) Quantitative analysis of disorganization of E-cadherin. The degree of disorganization of E-cadherin was categorized in four groups. Every visible crypt structure was analyzed and assigned to one of the categories for which examples are given below. Data was collected from two individual experiments (n = 2, at least 28 pictures per sample were analyzed) and blinded analysis of each experiment was performed by two individual persons. Significance was tested by using the Student’s t test and values refer to samples treated with toxin only (ns = not significant, *p < 0.05, **p < 0.01, ***p < 0.001).

Finally, the effect of VER on intoxication of stem cell derived induced human intestinal organoids (iHIOs) with CDT was investigated. The iHIOs are derived from hair sheet keratinocyte cultures from a healthy donor. After cellular reprogramming towards induced pluripotent stem cells, intestine organoids were generated in a stepwise differentiation protocol. These organoids display basic characteristics, such as crypt-like structures and architecture of a polarized intestinal epithelium, of human intestine tissue, containing both epithelial and non-epithelial cell types^46–48^. Therefore, iHIOs are also referred to as human miniguts. Within the binary clostridial toxins, CDT is of particular medical interest as it contributes to a more severe course and enhanced reoccurrence of *C. difficile* associated diseases^1^. Upon intoxication of miniguts with CDT, cytosolic CDT uptake was detected within epithelial cell compartments (Fig. 7a). Subsequently, less F-actin was detected in cells of CDT-treated miniguts compared to untreated miniguts. However, VER decreased CDT-mediated F-actin destruction and cortical F-actin was clearly more preserved and resembled more to the structure in untreated control organoids. Moreover, a clear distribution/organization of the adhesion protein E-cadherin mainly at the cortex of cells was observed in control miniguts whereas in CDT-treated organoids E-cadherin was more diffusely distributed and clustering of E-cadherin became obvious (Fig. 7a). VER-treatment led to reduced clustering and E-cadherin maintained its cortical localization comparable to control organoids. Quantification of E-cadherin disorganization from pictures (Fig. 7b) showed that in untreated controls about 90% of the crypts were in the two lowest categories of E-cadherin disorganization. However, in the majority of crypts (about 70%) from miniguts treated with CDT alone 90–100% of E-cadherin was disorganized whereas in miniguts treated with CDT in the presence of VER fewer crypts were assigned to this category (about 30%) and were mostly referred to the two intermediary categories.

Taken together, we demonstrated that Hsp70 plays a role for the transport of the A-components of binary actin ADP-ribosylating toxins across endosomal membranes into the cytosol of mammalian cells. The results further suggest that the functional interaction with Hsp70 during the intracellular membrane transport is specific for bacterial ADP-ribosylating toxins. Finally, we demonstrated the protective effect of an Hsp70 inhibitor on CDT intoxication of human miniguts, complex organoids displaying the architecture of the human intestine.

## Discussion

We identified Hsp70 as a novel interaction partner of clostridial binary actin ADP-ribosylating toxins *in vitro* and in living mammalian cells and showed that Hsp70 facilitates the trans-membrane transport of their enzyme components into the cytosol. These are the first toxins for which a contribution of Hsp70 during cellular uptake was observed and the results substantially extend our previous findings that Hsp90, Cyp40 and FKBP51 are necessary for efficient translocation and/or refolding of enzyme components of these toxins across endosomal membranes. Cyp40, FKBP51 and Hsp70 are co-chaperones of Hsp90, which bind to specific motifs like the MEEVD motif in Hsp90 with their corresponding tetratricopeptide repeat (TPR) motif^37, 38^. Thus, these host cell factors can form Hsp90-containing multi-chaperone complexes that facilitate folding and activation of client proteins in a concerted manner. In this regard it is interesting that *in vitro* the enzyme subunit (DTA) of diphtheria toxin, another ADP-ribosylating toxin, binds to a cytosolic Hsp90-containing protein complex and that the pH-dependent transport of DTA across endosomal membranes is facilitated by Hsp90 *in vitro* ^49^ and by Hsp90 and Cyps in living cells^50^. The activation cycle of steroid hormone receptors by an Hsp90-containing multi-chaperone complex is well characterized^36^. Remarkably, Hsp70 is one of the earliest chaperones interacting with the steroid hormone receptor transferring it to the Hsp90 machinery. The PPIases bound to Hsp90 determine affinity for the client protein and the ATPase activity of Hsp90^36^. In this context, our recent finding that Hsp70 is also involved in the transport of the enzyme component of iota toxin across membranes into the cytosol^33^, supports the hypothesis that the membrane translocation of ADP-ribosylating toxins might be facilitated by a multi-chaperone complex in cells similar to the steroid hormone receptor activating complex.

Here, we demonstrate that requirement of Hsp70 is not restricted to uptake of the iota toxin but also applies to the uptake of clostridial C2 and CDT enterotoxins. As reported recently for Ia, the C2I and CDTa directly bound to Hsp70 as well as Hsc70 as determined by dot blot analysis and binding is obviously enhanced if the enzyme components are in their denatured i.e. unfolded conformation. This finding is plausible since Hsp/Hsc70 is designed to bind unfolded client proteins^51^ and the enzyme components have to be at least partially unfolded to translocate through the narrow trans-membrane pores formed by their B-components (inner diameter ~20–40 Å^18^) into the cytosol^17^. By inhibition of Hsp70 activity with specific pharmacological inhibitors we demonstrated that Hsp70 is functionally involved in cytosolic uptake of clostridial binary toxins. Investigating the pH-dependent toxin transport across cell membranes in an isolated manner revealed that VER and HA9 inhibit this step of toxin uptake. This seems plausible since the unfolded enzyme components might need the assistance of cellular protein folding helper enzymes such as Hsp70 for their transport across endosomal membranes and/or their refolding into the enzymatically active conformation in the cytosol.

Interestingly, all identified bacterial toxins that require Hsp90/PPIases for their cellular uptake are ADP-RTs^32, 49, 52–54^. Toxins with different enzyme activities do not require Hsp90/PPIases for their cytosolic uptake, as demonstrated for the *C. difficile* glycosyltransferases TcdA and TcdB^27, 29^ or the protease lethal toxin of *B. anthracis* ^50^. However, fusion toxins artificially containing an ADP-RT domain instead of their native enzyme activity^31, 50^ as well as the isolated ADP-RT domain TccC3hvr from the *P. luminescens* toxin PTC3^55^ require Hsp90, Cyps and FKBPs for their uptake into the cytosol. This supports the hypothesis of a novel Hsp90/PPIase-dependent membrane transport mechanism which is specific and selective for ADP-ribosylating toxins. The results obtained in the present study also provide evidence that requirement of Hsp70 might be specific for ADP-RTs and might play no role for the cellular uptake of bacterial protein toxins, which are not ADP-RTs, since VER only inhibited intoxication with ADP-RT domains but not with glycosyltransferase and deamidase domains. Recently an involvement of Hsp90 for efficient uptake of clostridial neurotoxins (CNTs) with protease activity into cells was described^56^. However, requirement of Cyps and Hsp70 was not evident for CNT uptake suggesting that another Hsp90 complex different from the one required for the membrane transport of ADP-RTs facilitates translocation of CNTs. These results further support the hypothesis of a common Hsp90/Hsp70/PPIase-dependent translocation mechanism specific for ADP-RTs.

Fluorescence-based immunostaining revealed that less C2I protein was detectable in the cytosol upon inhibition of Hsp70 activity, suggesting that fewer C2I molecules translocated across endosomal membrane thereby remaining in endosomes. This conclusion was also supported by experiments employing a pharmacological inhibitor of proteasomal degradation. Under such conditions, the amount of cytosolic C2I was also reduced when Hsp70 and additionally the proteasomal degradation were inhibited suggesting that the reduced amount of C2I in the cytosol was not due to rapid degradation of misfolded C2I molecules upon Hsp70-inhibition. Moreover, by exploiting the PLA technology, the interaction between C2I and Hsp70 was shown for the first time in cells. Single molecule interactions in cells are difficult to visualize due to detection limits. The PLA technology overcomes this limitation due to an amplification process^57^, therefore enhancing the signal immensely.

However, whether Hsp70 is also involved in refolding of the translocated ADP-RTs during and/or after their trans-membrane transport could not be determined from conducted experiments and remains to be investigated. Also the precise molecular mechanism underlying the interaction between Hsp70 and the ADP-RT subunits is still unclear. The mechanism how Hsp70 facilitates membrane translocation of other proteins, e.g. of polypeptides into mitochondria, is described as entropic pulling^58^. This means that binding to the translocating polypeptide at the *trans*-side of a pore enhances unidirectional transport. Moreover, Hsp70 actively pulls the polypeptide through the pore due to conformational changes facilitated by ATP hydrolysis. Hydrolysis of ATP is also required to close the substrate binding pocket and thus for tight binding of client proteins. VER binds to the ATP binding pocket of Hsp70, Hsc70 and Grp78 and therefore interferes with tight binding and pulling of the client substrate. The recently described HA9 targets the substrate binding domain and also displays an improved specificity only for Hsp70 and not targeting Hsc70 or Grp78^33^. Our results indicate that both domains of Hsp70 are important for membrane translocation of clostridial enzyme components into the cytosol. Thus, first the unfolded translocating enzyme component has to bind to the substrate binding domain and then ATP hydrolysis can lead to tight binding and possibly to pulling of the enzyme component into the cytosol.

The importance of Hsp70 activity for efficient intoxication with CDT also became obvious in a human intestinal organoid model. This stem cell derived organoid model system is able to mimic a complex human gut structure that outperforms previous single cell model systems as it displays essential characteristics of complex mid-/hindgut structures like tight junctions or different epithelial and non-epithelial cell types^46^. Recently, advantages of such an organoid model system for dissection of basic disease mechanisms, e.g. infections with *Helicobacter pylori* were demonstrated^59^.

VER not only protected cells but also the organoid tissue structure from intoxication with CDT. VER reduced the depolymerization of F-actin induced by CDT and led to a preserved integrity/structure of miniguts displayed by F-actin and E-cadherin staining. The adhesion protein E-cadherin is connected to the actin cytoskeleton and therefore crucial for epithelial morphology and barrier function in the intestine^60^. During *C. difficile* infection the loss of epithelial barrier enables bacteria and also bacterial toxins to invade deeper into the tissue most likely supporting an improved colonization and persistence of *C. difficile* in the gut^1^. The disorganization of E-cadherin was also described upon intoxication of human intestinal organoids with TcdA, one of the two main toxins of *C. difficile* that glycosylate small Rho GTPases, which are important regulators of the actin cytoskeleton^61^. Moreover, loss of epithelial barrier function due to infection of miniguts with a TcdA- and TcdB-producing *C. difficile* strain was reported. In conclusion, human miniguts represent a highly attractive model to investigate the mode of action of clostridial enterotoxins and pharmacological inhibitors as we demonstrated for the protective effects of VER on intoxication of miniguts with CDT.

Finally, our results might be a starting point for development of novel therapeutic strategies to prevent and/or cure diseases associated with ADP-ribosylating bacterial toxins such as diphtheria, cholera, pertussis and enteric diseases. The membrane translocation of the enzyme subunits of such toxins into the host cell cytosol is a critical step in toxin uptake since the substrate molecules of ADP-ribosylating toxins resides in the cytosol. Thus, if the membrane translocation can be inhibited by specific pharmacological inhibitors of host cell factors like Hsp70, the cytotoxicity and therefore probably the clinical symptoms could be prevented. Therefore, supplementing antibiotic therapy with inhibitors of membrane translocation might offer a new therapeutic perspective in the fight against pathogenic (multi-) resistant bacteria producing ADP-ribosylating toxins.

## Materials and Methods

### Protein expression and purification, Synthesis of inhibitors

The following recombinant proteins were purified and activated as described before: C2I, C2IE387/389Q, C2IIa^13, 62^, C3bot^63^, Ia, Ib^22^, CDTa, CDTb (from *C. difficile* strain 196)^64^, His-TccC3hvr^65^, His-PaTox^GD^ ^43^, His-Afp18^44^, Hsp70/Hsc70^66^, FKBP12^67^ and PA63^68^. C2I, Ia, CDTa were biotin-labeled using sulfo-NHS-biotin (Pierce) and detected by streptavidin-peroxidase (Strep-POD, Sigma-Aldrich) in dot blot analysis. HA9 and NRLLLLTG were synthesized as described^69, 70^.

### Protein-protein interaction analysis using the dot blot system

Dot blot analysis was performed as described^31^. Purified proteins (Hsp70, Hsc70, FKBP12, C3bot) were spotted onto a nitrocellulose membrane in decreasing concentration (serial dilution, starting concentration 1 µg) via vacuum aspiration using the dot blot system. The transfer of proteins was confirmed by Ponceau S staining. Afterwards the membrane was blocked with 5% skim milk powder in PBST. The membrane was cut and probed with enzyme components (200 ng/ml) either in their native or denatured condition or with PBS for control for 1 hour. Denaturing of enzyme components was performed by pre-incubation with 6 M guanidine hydrochloride (GdmCl), 2 mM DTT and 30 mM Tris denaturation solution. After extensive washing the bound enzyme components were detected by Strep-POD using the ECL-system (Merck Millipore). Denaturation/unfolding of C2I was confirmed by monitoring enzyme activity (described below) as well as tryptophan fluorescence scan where tryptophan in C2I samples, incubated in PBS (control) or GdmCl solution, was excited at 280 nm and emission was detected from 300–500 nm (300–400 nm are shown) by Tecan Infinite M1000 Pro Reader using 96-well half area plates (Greiner Bio One).

### Cell culture and intoxication experiments

Vero (African green monkey kidney) and HeLa cells (from DSMZ, Braunschweig, Germany) were cultured in MEM (Invitrogen) plus 10% fetal calf serum (Invitrogen), 1.5 g/l sodium bicarbonate, 1 mM sodium pyruvate, 2 mM L-glutamine and 0.1 mM non-essential amino acids at 37 °C and 5% CO_2_. For reseeding, cells were detached with trypsin and no more than 25 reseeding cycles were performed. For intoxication experiments, cells were seeded in 96-well culture dishes. The specific inhibitors HA9 (inhibitor of the substrate binding domain of Hsp70), VER-155008 (inhibitor of the ATP-binding site of Hsp70, Hsc70 and Grp78, purchased from Tocris Bioscience), radicicol (inhibitor of ATP-binding site of Hsp90, purchased from Sigma-Aldrich) or bafilomycin A1 (inhibitor of v-ATPase, purchased from Santa Cruz Biotechnology) were added 30 min prior to toxin application to analyze the effect on the course of intoxication in the presence of these inhibitors. After given incubation periods at 37 °C and 5% CO_2_ with the respective toxin, pictures were taken using a Zeiss Axiovert 40CFI microscope with a Jenoptik progress C10 CCD camera. The morphological changes (i.e. cell rounding), which serve as a specific endpoint of intoxication, were analyzed and the percentages of rounded cells were determined from the pictures using ImageJ as described earlier^29^. Materials for cell culture were purchased from TPP Techno Plastic Products. The proteasome inhibitor MG132 was purchased from Sigma-Aldrich.

### Sequential ADP-ribosylation of actin in lysates from toxin-treated cells

ADP-ribosylation was analyzed as described^29^. After incubation with C2 toxin in the presence or absence of inhibitor, cells were lysed by freezing and scraped off in 25 µl ADP-ribosylation-buffer plus complete protease inhibitor (Roche). Lysates were incubated with 10 µM Biotin-NAD^+^ (Trevigen) plus 300 ng C2I at 37 °C for 30 min. SDS-PAGE and Western Blot were performed. The *in vitro* ADP-ribosylated, i.e. biotin-labeled, actin was detected by Strep-POD using the ECL system.

### *In vitro* enzyme activity and binding assay

Vero cell lysate (20 µg of protein) was pre-incubated with respective inhibitors or left untreated for control. C2I, Ia or CDTa and 10 µM biotin-NAD^+^ were added for 20 min at 37 °C. ADP-ribosylated, i.e. biotin-labeled, G-actin was detected by Western blotting. For binding analysis, Vero cells were pre-incubated with respective inhibitors or left untreated for control. Cells were cooled to 4 °C and C2, iota or CDT toxin were added for 30 min. Cells were washed with cold PBS three times and lysed. Cell-bound C2, iota or CDT toxin was detected by analyzing the ADP-RT activity of the cell-associated C2Ia, Ia and CDTa *in vitro* as described above.

### Toxin translocation assay

The toxin translocation assay was performed as described^13^. In brief, Vero cells were incubated with BafA1 for 30 min to inhibit normal toxin uptake. Cells were cooled down to 4 °C to allow binding of the subsequently added toxin. Cells were then challenged with warm acidic medium to facilitate translocation of the A-components through B-component pores directly across the cytoplasmic membrane into the cytosol of the cells. For control, toxin treated cells were challenged with neutral medium. Morphological changes were analyzed as specific endpoint of intoxication. To analyze the effect of VER and HA9 on membrane translocation, cells were pre-incubated with the respective inhibitor in parallel to BafA1 incubation.

### Immunofluorescence

For immunofluorescence experiments, cells were fixed by freezing methanol for 15 min at RT, permeabilized with Triton-X 100 (0, 4% in PBS) for 5 min at RT, treated with Glycin (100 nM) in PBS for 2 min at RT and blocked with 10% FCS in PBST for 1 h at 37 °C. Samples were incubated with C2IN antibody (rabbit) (1:1500 diluted in 10% FCS in PBST) for 1 h at 37 °C. After washing, fluorescence-labeled secondary antibody anti-rabbit 647 (donkey) (Thermo Fisher Scientific) was added (1:500 diluted in 10% FCS in PBST) for 1 h at 37 °C. Fluorescence imaging was performed using the iMic Digital Microscope (FEI Munich) using the Live Acquisition 2.6 software (FEI Munich) and processed with ImageJ 1.4.3.67 software (National Institutes of Health, Bethesda). The average cytosolic C2I labeling was determined from plot profile lines across individual cells in background-corrected images (Image J 1.4.3.67, Fig. 5a and b). Plot profile data were exported into Matlab V9.0 (The MathWorks) for the identification and exclusion of endosomal peaks (see Fig. 5c). The area under the curve (AUC) of plot profile minima was taken as a measure of cytosolic labeling (see blue circles and area in Fig. 5c). Minima were determined by the slope at the roots of the first derivative of spline filtered plot profiles. AUCs were corrected for the length of the plot profile line (AUC/length) to obtain the cytosolic C2I density (Fig. 5c).

### Analysis of protein interaction in cultured cells by Duolink using PLA technology

Vero cells were pre-incubated with or without respective inhibitor and then C2 toxin was added for 1 h at 37 °C. Cells were fixed with methanol and permeabilized. Incubation with rabbit anti-C2IN serum and mouse anti-Hsp70 antibody (Enzo Life Sciences) was performed. Subsequently, the PLA assay was performed according to the manufacturer’s protocol (Duolink using PLA technology, Sigma-Aldrich). In brief, cells were incubated with two secondary antibodies (anti-rabbit for detecting C2I and anti-mouse for detecting Hsp70) that each contain a specific oligonucleotide sequence. Moreover, oligonucleotides that are partially complementary to the probes coupled to the antibodies were added. Only if both antibodies got in close proximity, the complementary oligonucleotides could form a ring structure and then could be ligated by an added ligase to a circular DNA. Subsequently, a polymerase is added that amplifies the circular DNA by rolling-circle amplification. The samples were probed with fluorescence labeled oligonucleotides that were complementary to the amplification product and therefore bind to these products. In conclusion, a fluorescence signal, enhanced by amplification, only occurs when C2I and Hsp70 are in close proximity i.e. interact with each other. The PLA signals were counted form fluorescence pictures and given as signals per cell. PLA signals were counted form fluorescence pictures using a computer-vision algorithm based on OpenCV.

### Intoxication of induced human miniguts

The use of human material in this study has been approved by the ethical committee of the Ulm University (Nr. 0148/2009) and Tübingen University (638/2013BO1) and in compliance with the guidelines of the Federal Government of Germany and the Declaration of Helsinki concerning Ethical Principles for Medical Research Involving Human Subjects. A healthy proband gave a written informed consent. Human intestinal organoids were incubated in matrigel and medium. For intoxication experiments, organoids were placed in a 24-well plate. Prior to intoxication, organoids were pre-incubated with 60 µM VER or left untreated for control for 30 min at 37 °C. Then, 500 ng/ml CDTa and 1000 ng/ml CDTb were added for 3 h at 37 °C. After washing organoids were released from matrigel, washed again and then fixed by 4% PFA in 10% sucrose solution for 20 min at RT. After washing and incubation in 25% sucrose solution over night at 4 °C, organoids were placed in cryomolds, embedded in tissue-tek OCT compound and frozen in liquid nitrogen. Frozen sections (8 µm) were made and dried over night at RT. After rehydration, samples were blocked (10% goat serum), permeabilized (0.2% Triton X100 in PBS) and treated with antibodies against CDTa and E-cadherin over night at 4 °C. Fluorescence-labeled secondary antibodies in 5% goat serum and 0.1% Triton X100 in PBS were added for 1 h at RT. Moreover, nuclei were stained with DAPI and F-actin with phalloidin-FITC. Confocal microscopy was performed. Quantitative analysis of the effect of CDT on E-cadherin organization was performed by determining four categories of degree of disorganization of E-cadherin structure. Examples for the different categories are given. Data were collected from two individual experiments (n = 2, at least 28 pictures per sample were analyzed) and blinded analysis of each experiment was performed by two individual persons.

### Reproducibility of the experiments

All experiments were conducted independently at least two times and results from representative experiments are shown in figures if not indicated otherwise. Western blot panels were cropped and recombined for presentation purposes only and corresponding protein bands were originally detected on the same membrane and X-ray film.
